# A Review of Pediatric Critical Care in Resource-Limited Settings: A Look at Past, Present, and Future Directions

**DOI:** 10.3389/fped.2016.00005

**Published:** 2016-02-18

**Authors:** Erin L. Turner, Katie R. Nielsen, Shelina M. Jamal, Amelie von Saint André-von Arnim, Ndidiamaka L. Musa

**Affiliations:** ^1^Asante Rogue Regional Medical Center, Pediatric Hospital Medicine, Medford, OR, USA; ^2^University of Washington, Seattle, WA, USA

**Keywords:** pediatric critical care, resource-limited setting, Ebola epidemic, millenium development goal 4, resource allocation

## Abstract

Fifteen years ago, United Nations world leaders defined millenium development goal 4 (MDG 4): to reduce under-5-year mortality rates by two-thirds by the year 2015. Unfortunately, only 27 of 138 developing countries are expected to achieve MDG 4. The majority of childhood deaths in these settings result from reversible causes, and developing effective pediatric emergency and critical care services could substantially reduce this mortality. The Ebola outbreak highlighted the fragility of health care systems in resource-limited settings and emphasized the urgent need for a paradigm shift in the global approach to healthcare delivery related to critical illness. This review provides an overview of pediatric critical care in resource-limited settings and outlines strategies to address challenges specific to these areas. Implementation of these tools has the potential to move us toward delivery of an adequate standard of critical care for all children globally, and ultimately decrease global child mortality in resource-limited settings.

## Introduction

Fifteen years ago, world leaders convened at the Millennium Summit of the United Nations to set key goals to be achieved by 2015. Specific to pediatrics, Millenium Development Goal 4 (MDG 4) sought to reduce under-5-year mortality rates by two-thirds. Despite a rapid reduction in under-5-year mortality for some countries, the progress in sub-Saharan Africa and Southeast Asia remains insufficient to meet the goal (1). Only 27 of 138 developing countries are expected to achieve MDG 4. The five countries with the highest under-5-year mortality rate in 2013 were India, China, Pakistan, Nigeria, and the Democratic Republic of the Congo (1).

The majority of childhood deaths in these settings result from preventable and reversible causes. Beyond the neonatal age, these include lower respiratory tract infections, malaria, diarrheal disease, meningitis, and nutritional deficiencies (2, 3). While ~10–20% of sick children will be referred to a hospital, the delay in recognition, late presentation, lack of resources, and illness severity make the first 24 hours of hospitalization the most vulnerable period with a third of patient deaths occurring during this time (4).

In addition to preventative care and nutritional support, the development of effective pediatric emergency and critical care services in resource-limited countries can substantially reduce global mortality in children under 5 years (5, 6). Resource-limited settings, or areas where the capability to provide care for life-threatening illness is limited to basic health care resources, pose a specific challenge to the development and sustainability of critical care services (6).

## The 2014 Ebola Epidemic

The Ebola outbreak that ravaged West Africa provided an example of the considerable need for better healthcare infrastructure and supportive care of critically ill patients in resource-limited settings. This is not a new revelation: natural and human-generated events, such as disasters and wars, produce acute and unpredictably large numbers of critically ill patients around the world. Recent historical examples include the severe acute respiratory syndrome (SARS) and H1N1 influenza epidemics. As of June 2015, the overall fiscal impact of the Ebola epidemic has been estimated to be upwards of $30 billion, while only $7.1 billion has been pledged thus far (7, 8).

**Figure 1:**
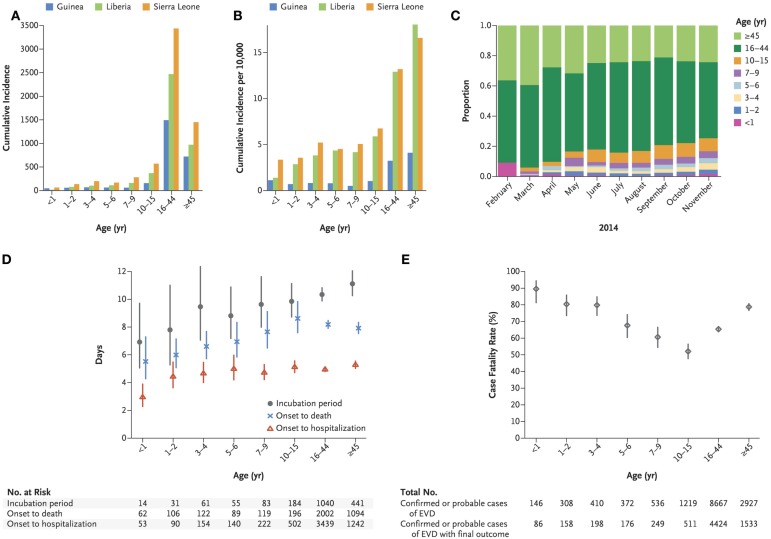
**Age-group-specific incidence of Ebola virus disease in West Africa, incubation period, intervals from onset to death and onset to hospitalization, and case fatality rate. (A)** shows the cumulative incidence of confirmed and probable cases of Ebola virus disease (EVD) according to age group and country. **(B)** shows the cumulative incidence of confirmed and probable cases per 10,000 population according to age group and country. **(C)** shows the overall age distribution for confirmed and probable cases according to month of symptom onset. **(D)** shows the estimated average incubation period, the interval between symptom onset and death, and the interval between symptom onset and hospitalization according to age among persons with confirmed or probable EVD [with vertical lines indicating 95% confidence intervals (CIs)]. The numbers represent the sample sizes in each age group. **(E)** shows the estimated case fatality rate according to age among persons with confirmed or probable EVD (with 95% CIs), with representation of the total number of confirmed or probable cases of EVD cases in each age group and the total number of confirmed or probable cases of EVD for which there was information on the final outcome in each age group. Copied with permission from: WHO Ebola Response Team, et al. (11)

According to the latest Center for Disease Control (CDC) data, Guinea, Liberia, and Sierra Leone were the hardest hit with the Ebola epidemic. Suspected, probable, and confirmed Ebola infections have surpassed 25,000, with over 11,000 deaths (9). Historically, children have been underrepresented in Ebola virus disease (EVD) outbreaks due to outbreak dynamics and societal structure (10). Reliable estimates of affected pediatric patients are limited, though a recent report by the WHO Ebola Response Team estimated 1400 EVD-related deaths in pediatric patients (7). Case-fatality rates were highest in patients <5 years. Children demonstrated relatively shorter incubation times and more rapid progression to death, emphasizing the need for early recognition and prompt access to care (see Figure 1) (11).

The Ebola outbreak highlighted the fragile state of health care systems in resource-limited settings. There is an urgent need for a paradigm shift in healthcare delivery and outcomes of critical illness in these settings. What Ebola patients need most stems from the foundation of critical care and public health: early recognition, supportive care, fluid resuscitation, and electrolyte management within an infrastructure organized to support hygiene, sanitation, isolation practices, safe handling, and transportation services (12). Unfortunately, patients in Western Africa died due to the inability of health care systems to provide this foundation (13). The current emphasis on disease specific as opposed to care delivery-based programs in resource-limited settings makes the provision of efficient and effective healthcare challenging, clearly shown by this Ebola outbreak. Improvement in the fundamentals of critical care medicine can and should be instrumental in helping make these urgently needed health care delivery shifts happen.

## Development of Pediatric Critical Care Medicine

### History

Unlike other medical specialties defined by organ system, disease process, or procedure, critical care has struggled to establish its identity. The concept of frequent monitoring of vital signs in postoperative patients evolved in Germany and the US between 1890 and 1910. The poliomyelitis epidemic of the 1920–1950s provided momentum for the development of modern respiratory care and intensive care medicine. Polio wards represented the first respiratory intensive care units with specialized nursing and medical staffs providing 24 hour care for patients with organ-system failure (14).

The history of pediatric critical care dates back to 1955, when the first unit was opened at the Children’s Hospital of Goteborg in western Sweden. Physicians used their knowledge and skills acquired in managing polio patients to provide intensive care services, including mechanical ventilation to newborns with respiratory distress syndrome, postoperative children with sepsis, and cases of severe pneumonia. It was not until 1971 that the first pediatric intensive care unit (PICU) dedicated to older infants and children was opened in Toronto, Canada. Subsequent decades brought many innovations in care, changes in care models, and maturation into a recognized subspecialty with accredited fellowship training programs.

### Pediatric Critical Care in Resource-Rich Settings

The definition of a patient in need of critical care has evolved over the years. To allow for policy development of new PICUs across the US, the American Academy of Pediatrics published PICU admission criteria in 1999 that were reaffirmed in 2008 (15). As one could expect, the criteria were broad and deliberately vague: (1) severe, life-threatening, or unstable cardiovascular, neurologic, hematologic/oncologic emergencies, (2) endocrine/metabolic, gastrointestinal, renal and/or multisystem disease, (3) postoperative patients requiring frequent monitoring and potentially requiring intensive intervention, and (4) conditions that necessitate the application of special technologic needs, monitoring, complex intervention, or treatment, including medications associated with the disease that exceed individual patient care unit policy limitations. The Society of Critical Care Medicine (SCCM) guidelines recommend only that “ICU admission criteria should select patients who are likely to benefit from ICU care (16).” Limited information exists to support which patients fall into this category. This variation in admission practices makes quantification of critical illness difficult (17).

Organizational details of intensive care units vary considerably. The presence and utilization of PICUs, intermediate units, step-down units, and specialty-specific units differ from country to country, state to state (18). Composition of PICU team members has not been standardized and is often driven by the availability of resources in that institution. Most team members (physicians, nurses, respiratory therapists, pharmacists, dieticians, etc.) have completed some form of pediatric ICU-specific training.

### Pediatric Critical Care in Resource-Limited Settings

Understanding the true burden of critical illness in resource-limited settings is challenging, hindering both local and global appreciation. As outlined by Murthy et al. (19), three general approaches can be used to try to estimate the global burden of critical illness. The first includes counting patients admitted to ICUs around the world, which would provide an underestimate, given the lack of ICUs in resource-limited settings. The second method extrapolates from resource-rich countries’ epidemiology, which is fraught with its own error as discussed above. The third approach would use the assumption that all deaths occurring in a region involved critical illness at some stage of the illness, likely leading to an overestimate. The lack of a reliable method has led to substantial variation in disease burden estimates in resource-limited settings. During the 2009 H1N1 pandemic, two independent groups estimated regional H1N1-associated mortality and found great variation, in particular in the African region (20–22). The 2014 EVD outbreak was difficult to quantify, leading to discrepancies in numbers reported by WHO and CDC (9). The first EVD outbreak in Congo in 1976 was halted in part by clearly defining cases and contacts, highlighting the importance of understanding epidemiology in resource-limited settings (23).

Application of illness severity scores developed in resource-rich countries, such as PRISM and PIM, have been used to predict mortality and thereby the burden of critical illness (24). A variety of scores have been evaluated in resource-limited settings with conflicting results. Although PIM and PIM2 were validated at tertiary care PICUs in India and Pakistan (25, 26), both PRISM and PIM had poor predictability in other centers in India and South Africa (27, 28). Scores that are reliably applicable in all resource-limited settings are lacking, making definition of critical illness and estimation of burden of disease more challenging.

The most recent Ebola outbreak highlighted the deficiency in the ability to provide basic acute and critical care services in resource-limited settings. Patients with illnesses that could be cared for in an intermediate or step-down unit in one part of the world may require PICU-level care in another. Therefore, application of aforementioned AAP admission criteria could be done with resource-driven adjustments. SCCM guidelines, as stated above, weigh heavily here with attention on appropriate allocation of care.

Organizational details of pediatric ICUs in these settings are lacking. Team composition is often limited to a general pediatrician, registrar/trainee, and a handful of senior-level nurses. Pediatric intensivists, respiratory therapists, pharmacists, or dieticians are often not available (6). The 2006 World Health Report stated that there was a global shortage of 4.3 million doctors, midwives, nurses, and support workers. This shortage can be explained by (1) lack of health staff training, (2) subsequent loss to higher paying jobs within a trainee’s country after training is complete, or (3) emigration to richer countries. While the latter causes are attributed to bureaucratic infrastructure around appropriate financial compensation and are difficult to correct on a mass scale, the lack of healthcare staff training can be ameliorated. With the recent Ebola outbreak, a common assumption was that the lack of material resources had been the dominant barrier to clinical care. However, in some areas, basic supplies such as intravenous catheters, fluids, and oral electrolyte replacement solutions were often readily available, but the lack of trained personnel to administer them was the true limitation (13).

## What has been Done to Address Millenium Development Goal 4

With unacceptably high mortality rates in children highlighted both during times of epidemics and in times of relative steady state, the WHO and UNICEF strategies for reducing mortality in children under 5 years initially focused on outpatient management and primary care (29). The first WHO guidelines, the Integrated Management of Childhood Illness (IMCI), are an evidence-based strategy for assessing and treating sick children in the ambulatory care setting. Even with the focus on primary care, these guidelines assume that sick children will be referred to a hospital for escalation of care if needed (29). With appropriate IMCI use, 16–46% of children will require referral to a hospital (30–33).

The need for excellence in clinical care does not end simply with the referral. In an urban population in Guinea-Bissau, 45% of children under 5 years were hospitalized, and the in-hospital mortality was 12% in 2007 (34), compared with an inpatient mortality of 0.84% in the US in 2009–2010 (35). Challenges in presentation, triage, initial management, and admission to the hospital setting prompted guideline development and interventions for improved patient outcomes. Nolan et al. demonstrated that more than half of children were undertreated or inappropriately treated with antibiotics, fluids, or oxygen in 21 hospitals across seven countries in Asia and Africa (36).

In response to the need for improved in-hospital management, the WHO acute hospital care guidelines, termed the Emergency Triage, Assessment, and Treatment (ETAT) guidelines, were developed. This training program is designed to prioritize care for children who need urgent resuscitation and hospital admission. In Malawi, uptake and implementation of the ETAT guidelines streamlined the delivery of care and reduced hospital mortality by half (10–18% down to 6–8%) (4). However, the first 48 hours of admission were identified as the vulnerable period where skills beyond emergency care were needed. ETAT-plus admission care (ETAT+) was developed in Kenya to address this (37). Despite some positive results of ETAT^+^ implementation and further dissemination of these guidelines and training, reports indicate significant variation in uptake of best practices (36, 38–41).

Various other hospital-based initiatives have allowed for further intervention of pediatric critical care in resource-limited settings. The WHO Integrated Management of Adult and Adolescent Illness (IMAI) program, a sister initiative to IMCI, created a comprehensive manual for the care of hospitalized patients by clinicians in district hospitals, which includes sections on severe illness management (42). The European Society of Intensive Care Medicine produced sepsis management guidelines targeting resource-limited settings (43). These largely expert opinion-based documents fill major gaps in management guidelines but require further research to provide missing evidence and refine best-practice recommendations.

In addition to the development of guidelines, training modules and various curricula have been developed to build further educational capacity. Courses, such as advanced pediatric life support (APLS and PALS), often in partnership with a mission organization or supporting institution, have been widely implemented (44). Nursing, physician, and paramedic pediatric emergency curricula have been successfully implemented in Ethiopia and Ghana (45).

The WHO also produced the *Pocket Book of Hospital Care for Children*, providing clinical guidelines for physicians and nurses delivering pediatric hospital care in resource-limited settings (46). These guidelines have been adapted to local needs and increasingly adopted by Ministries of Health around the world (47). An online and postal survey of almost 100 low- and middle-income countries reported that 25% have successfully implemented and used the Pocket Book in their clinical care, and 40% showed at least partial implementation (48, 49). Adherence and local adaptation of WHO guidelines for treatment of severe malnutrition in South Africa significantly decreased mortality by 7–15% in various hospital settings (50).

However, recent evidence from Kenya showed that health workers at the hospital level were unable to provide appropriate care for severely ill newborns or children with inadequacies in key tasks, such as prescription of antibiotics and feeds, even when resources are available (51). In attempting to improve these deficiencies, the level of engagement of senior and particularly mid-level clinical managers was important (52). These studies show that the simple availability of either WHO or national guidelines alone do not improve hospital care for children as hoped. It appears that broader, more system-oriented interventions addressing the many important influences on provider or user behavior are needed (53). A recent multifaceted approach addressing deficiencies in knowledge, skills, motivation, and organization of care utilizing face-to-face feedback of performance, supportive supervision, and provision of a local facilitator resulted in more sustained improvements of pediatric care in Kenya (38).

## The Cost of Critical Care

Critical care is complex and expensive, regardless of where it is practiced. Daily ICU costs in Indian private hospitals are similar to North America (54). However, in many resource-limited settings, the ICU provides basic rescue interventions for children and young adults who are ill with curable diseases. This is in contrast to the shift in practice of ICUs in high-income countries, which now admit and care for mostly complex patients with often incurable diseases during long hospitalizations (55). Provision of intensive care services may not seem rational or cost-effective in low-income countries, especially in those countries with the highest child mortality rates (56). However, a short duration of critical care that treats acute, life-threatening, curable illnesses affecting millions of young people worldwide may have a large impact on mortality (55).

The World Health Organization defines a “very cost-effective” intervention as one that costs less than the value of gross domestic product (GDP) per capita per disability-adjusted life year (DALY) (57). This includes not only many primary care and preventative interventions (58–60) but also management of childhood pneumonia and some surgical interventions (44, 61–63). Critical care costs include expenses for personnel, equipment, and consumables, as well as the impact of long-term morbidity of survivors. The higher costs of ICU care contribute substantially to the limitations of critical care infrastructure in resource-limited settings (18).

Some relatively inexpensive and simple critical care interventions dramatically improve patient outcomes, including the introduction of oxygen concentrators, oximetry monitoring, and supplemental oxygen in Papua New Guinea, which decreased pediatric pneumonia case fatality by 35% (64). Development of low-cost intensive care equipment, such as inexpensive ventilators (65, 66), and relocation of ICU equipment production into low- or low–middle-income countries to avoid expensive import from North America and Europe may help drive down the cost (67). Further evaluation of cost-effective interventions is needed to understand which ones are appropriate, feasible, and sustainable. In resource-limited settings, cost analyses would not only guide decision-making around resource allocation in the ICU, but also help determine health infrastructure and systems needs to address the long-term morbidity of ICU survivors (68).

## Ethics of Pediatric Critical Care in Resource-Limited Countries

The ethical issues of increasing emergency and critical care services in resource-limited settings remain challenging. The main areas of consideration include global justice, resource allocation, and local cultural preferences. Although a complete discussion of global health ethics is beyond the scope of this review, we will briefly discuss the major issues of relevance for critical care and refer readers to Simon Caney’s book, “Justice Beyond Borders” (69), for additional information.

### Global Justice

Arguments of global justice state that healthcare services are fundamental and universal human rights to be shared by the masses (19). The *just* distribution of healthcare services across all human populations remains a serious challenge (70, 71). One of the main arguments for the provision of critical care services in resource-limited settings lies within the definition of cosmopolitanism, which affirms three principles: the worth of individuals, equality, and the existence of obligations binding to all. Global disparities are highlighted during times of crisis, such as the Ebola outbreak, leading to outcries for humanitarian interventions (69). This implies that the responsibility and commitment of those from resource-rich regions is required to combat critical illness and strengthen health care infrastructure for those in resource-limited settings (69).

### Resource Allocation

The allocation of critical care services in the most deprived areas of the world must not seek to imitate the developed world (72). Riviello and colleagues have identified ways to address ethical dilemmas of resource allocation in resource-limited settings (73), including obtaining data on disease prognosis with and without ICU level care to understand its impact; development of guidelines to assist in such decisions in an open and honest manner; and creation of hospital policies on the use of critical care services (73).

The following examples highlight the ethical challenges that have arisen in resource allocation of critical care services. The 2010 Haiti earthquake, which killed 230,000 and displaced approximately 2 million, prompted the development of one of the first ever neonatal-PICUs in the country via non-governmental organization support. Limitation of care decisions were made in the setting of cardiac arrest or respiratory failure necessitating intubation in children with chronic disease. The delivery of quality ICU care was challenging, but was most successful and beneficial for otherwise healthy children who presented with early, acute, reversible disease (74). In South Africa, in an attempt to provide a reasonable process for fair and equitable utilization of scarce resources, the local PICU team at Red Cross War Memorial Hospital developed explicit admission exclusion criteria (Table 1) (75).

**Table 1:** **Specific exclusion criteria**.

Category	Specific exclusions	Comments
Futile care	A child who has been declared brain dead	The permanent vegetative state was not addressed in the admission criteria as this was more likely to be encountered as a problem following stay in the PICU
	A child who has had a cardiac arrest and has not reestablished a normal respiratory pattern, or who has fixed dilated pupils	<5% of children in this category survive the PICU admission with an acceptable neurological outcome
	The child who has suffered a head injury such that there is no chance of recovery from that injury	
Children with underlying lethal conditions	Children with burns >60% body surface area, where the surgical team are not able to guarantee that debridement and appropriate cover will happen within 24–48 h of admission	Based on data that if children are not debrided and grafted early on in the course of their burn management, they suffer a prolonged course with considerable pain, anxiety, and recurrent infection. The death rate in these children is also unacceptably high
	Children with chronic renal failure where there is no commitment to long-term dialysis	
	Children with severe and lethal chromosomal abnormalities (e.g., Edward syndrome or thanatophoric dwarfism)	
	Children with malignancies that are not responding to therapy	
	Children with inoperable cardiac lesions	
Children with currently poor outcomes	Children with established HIV infection. “Children with established HIV infection whose lives are in danger from AIDS-related diseases will not normally be considered for admission. A child who is successfully established on ARV, and where the reason for admission does not relate to the underlying disease and/or its therapy will be considered for admission.”	Based on data that despite the availability of ARV, only approximately 20% of children with HIV infection who were admitted to the PICU were known to be alive and on ARV 6 months later. These data have not changed since the availability of antiretroviral therapy
	Children with kwashiorkor	Based on a virtual 100% mortality in the ICU for these patients
	Children who have been in hospital wards for >5 days and are deteriorating despite appropriate therapy	Based on an extremely high mortality rate in this group of patients. The failure to respond to therapy suggests that they have underlying conditions that are not amenable to conventional therapy
	Children with severe adenoviral pneumonia who have not responded to appropriate therapy in the wards	Based on data showing that children with severe adenoviral infection requiring ventilation have a high mortality and very high morbidity from chronic lung disease
	Children with diagnosed severe metabolic disorders (e.g., maple syrup urine disease) for which established treatment programs in the hospital and community are not established	Based on the fact that these children have a very poor likelihood of reasonable outcome
	Children with acute hepatic failure, unless there is a reasonable likelihood that an acute transplant will be offered within the first 24–48 h of PICU admission	
	Children with complications of meningitis requiring ventilation (i.e., the requirement for ventilation is related to CNS disease rather than pneumonia)	
	Children with cardiomyopathy unresponsive to therapy, and where transplantation is not being considered	This does not apply to the time of acute, first presentation. It is very difficult to prognosticate at that stage. This comment applies to children who have previously been treated, and where there has been time to make an appropriate assessment of likely prognosis.

*Copied with permission from Argent et al. (75)*.

*ARV, antiretroviral drugs*.

### Family and Cultural Preferences

In addition to the difficulties with resource allocation, providing ICU level care while respecting family, cultural, and religious preferences can be challenging. Local belief systems greatly influence the approach to life and death circumstances and must be a part of conversations between providers and families. Decisions surrounding escalation of care need to be made in the context of potential financial impoverishment of the entire extended family, especially if the most likely end result is a child’s long-term morbidity or death (5, 8, 9). In many resource-limited settings, the provision of care follows a fee-for-service model (67, 76). Even for patients with a good prognosis, care may be limited if the family is unable to afford it or the hospital unable to absorb the cost (77). Incorporation of international guidelines into local practice and policy requires respecting local norms and consequences on patients’ family units (9).

## Critical Care Infrastructure and Capacity Building in Resource-Limited Settings

If the Ebola virus were to have appeared in high-income countries, there is little doubt that healthcare systems would have effectively contained and eliminated the disease with far lower case-fatality rates than those reported in West Africa over the past couple years. As mentioned above, the lack of adequate health care staff, resources, and systems required for the delivery of high-quality health care services contributed to the poor outcomes of the EVD epidemic in West Africa (78). Emergency care, including triage, is often one of the weakest parts of health systems in resource-limited settings. When triage is well organized, it saves lives in a cost-effective manner in a wide range of settings, patient populations, and systems (4, 79). Emergency and triage training (80, 81); transport training (82); simplified protocols, and treatment algorithms have all resulted in reduced morbidity and mortality of critically ill children (4).

The Ebola epidemic highlighted deficiencies in healthcare systems, emphasizing broad global inequalities. This epidemic should, therefore, not only lead to provision of critical aid in the short-term, but also lead to investment in creating systems that provide sustained, effective quality health care for all critically ill patients (83). This effort should focus on increasing the health care work force, available resources, and systems.

### Health Care Work Force and Education

**Figure 2:**
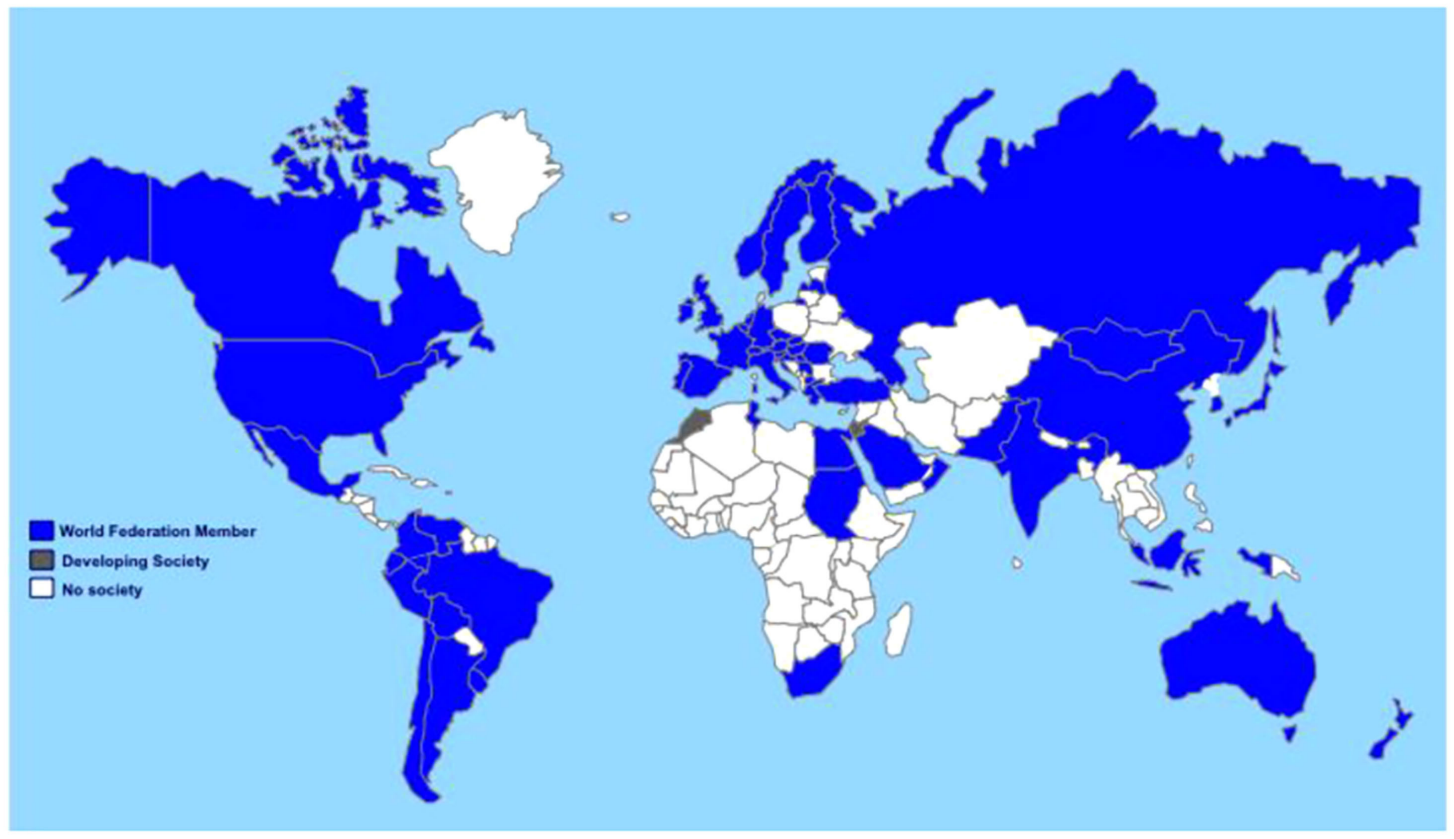
**Accurate numbers of critical care centers and services worldwide are unknown**. Membership in the World Federation of Societies in Intensive and Critical Care Medicine is used as an extrapolation of possible services worldwide, with countries illustrated where membership Societies are fully developed (blue), those where existing membership is developing a professional Society (gray), and countries without Federation members (white). Copied with permission from: Resource-Poor Settings: Infrastructure and Capacity Building Resource-Poor Setting: Infrastructure and Capacity Building: Care of the Critically Ill and Injured During Pandemics and Disasters: CHEST Consensus Statement (114).

The scarcity of health care workers in sub-Saharan Africa poses a serious challenge (see Figure 2). As an example, even before the Ebola outbreak, Liberia’s 4.3 million people were served by just 51 physicians (84). The dearth of physicians with training in pediatric emergency and critical care is even more significant. In Nigeria, 380 critical care trained nurses care for a population of over 140 million (85). Training opportunities in critical care in resource-limited settings are scarce, frequently requiring travel to high-income regions, which increases the risk of emigration of health care providers from low to high-resource countries, commonly referred to as “brain drain” (86, 87). Recognizing the impact of this phenomenon on workforce shortages, the WHO unanimously adopted the WHO Global Code of Practice on the International Recruitment of Medical Personnel in 2010 (88): this Code aims to promote ethical recruitment of health personnel to strengthen health systems and member states are discouraged from active recruitment of health workers from those countries faced with critical shortages of personnel. Unfortunately, the Code of Practice has not slowed the emigration of physicians from sub-Saharan Africa to the US (89). Solutions include posing limitations on active “poaching” by high-income countries, founding and recognition of national critical care societies (90), development of domestic training programs for healthcare staff linked to a commitment of service (73, 91), utilization of physician extenders or task-shifting (92), and building capacity through education initiatives.

#### Critical Care Education

Despite a long history of pediatric critical care training in resource-rich countries, substantial variation in duration, content, and assessment suggest that the optimum model for training remains unclear (93). The lack of a gold standard in training makes the design of a critical care curriculum in resource-limited settings much more complicated. Many countries have implemented local training programs with guidance from local experts; however, most of these programs lack oversight from a larger governing body or society, such as the American Board of Pediatrics, the Royal College of Physicians and Surgeons of Canada, or the Joint Faculty of Intensive Care Medicine of Australia and New Zealand. This leads not only to discrepancies in the provision of care but also to duplication of efforts in regions already struggling with scarce resources.

Attempts at creation of pediatric critical care training programs through international collaboration have begun in several places, including both Ecuador and Kenya.^1^ These partnerships seek to address the issues of implementation of an educational curriculum, the applicability to the local health care systems, diseases, and culture and the long-term sustainability. Critical care training can be further leveraged through visiting, temporary trainers from resource-rich countries, until local critical care capacity is self-sufficient and local trainers have been trained. Long-term success and sustainability of these initiatives remain to be seen.

Critical care education and training can also be supplemented with available free pediatric critical care resources. Some examples include Open Pediatrics^2^ and Pediatrics BASIC^3^. An online version of Pediatric Fundamental Critical Care Support^4^ is available; however, the minimum cost of $600 USD makes global access, particularly in resource-limited settings, prohibitive.

Simulation training provides another opportunity to engage learners regardless of language and cultural barriers and has been found especially useful in introducing primary triage and culturally sensitive treatments (94, 95). With the improvement and advancement in technology, the interactive delivery of healthcare over distance using technology such as video conferencing or telemedicine can increase access, improve outcomes, and reduce costs (96). In work done by Médecins Sans Frontières (MSF) in Somalia, introduction of telemedicine resulted in a change in case-management in 64% of children for which it was used and detection of a previously undetected life-threatening condition in 25% (97). However, the reliable application of this technology in resource-limited settings can be challenging. The MSF system demonstrated a median response time of 13 h making its utility in critical, time-sensitive cases, somewhat limited (98).

#### Task-Shifting

This practice consists of training non-physicians to substitute for critical care physicians. It has the ability to aid in the development and dissemination of guidelines and protocols to address the existing shortfall of limited ICU capacity and resources (18). In the local communities, community health workers can play a prominent role in early recognition and triage of severely ill children. Their efficacy in the lowest resource areas of the world (childhood mortality >30 per 1000 children) has been validated by Bang et al. who demonstrated an eightfold reduction in neonatal mortality with the administration of intramuscular (IM) antibiotics alone by village health workers trained in neonatal care and sepsis (99). They also serve a role in encouraging patients and families to seek care early at health care centers. Once in the hospital setting, appropriately trained nurses are required for successful integration of prevention and disease treatment (83).

Specialist nurses or anesthetic medical assistants trained in critical care are vital when physicians are scarce (72). From 1998 to 2008, MSF trained 24 nurses to become nurse anesthetists in Haiti with high completion and retention rates. They were essential in providing anesthesia for 330 procedures during the post-hurricane emergency in 2008 (100). Severe shortages of acute care and healthcare workers in rural Uganda prompted the development of an emergency mid-level provider training program. Nurses participated in a 2-year program. Outcomes of over 10,000 patients were evaluated and demonstrated lower than previously published regional mortality rates (101). A 2014 Cochrane review found evidence that shifting responsibility from physicians to adequately trained and supported nurses and/or community healthcare workers for managing HIV patients did not decrease quality of care, and in fact, may have decreased the numbers of patients lost to follow-up (102). The utility of task-shifting in critical care settings needs to be further explored before recommendations around its implementation can be made.

#### Critical Care Guidelines and Toolkits

Low-cost, high yield critical care in resource-limited settings can be provided using practice-support tools, such as guidelines, protocols, checklists, and standard order sets (103). A revised South African Triage Scale (SATS) for children was validated in multiple centers across South Africa with high sensitivity and negative predictive value to assist in early prioritization of pediatric patients seeking emergency care. It uses clinical signs along with a triage early warning score (TEWS) to assist in early recognition of acute illness, increase efficiency of patient discharges, all while promoting more effective use of hospital resources (104). Integration of emergency triage assessment and treatment (ETAT) guidelines into local practice and use of neonatal resuscitation practice through Helping Babies Breathe have shown decrease in childhood and neonatal mortality, respectively (4, 95, 105). During the H1N1 influenza pandemic, the WHO assembled a group of experts to generate a document addressing management of severe respiratory distress and shock in resource-limited settings (106). More recently, similar advice was produced by the WHO for the clinical management of novel coronavirus from outbreaks in 2012 in the Middle East (107).

**Figure 3:**
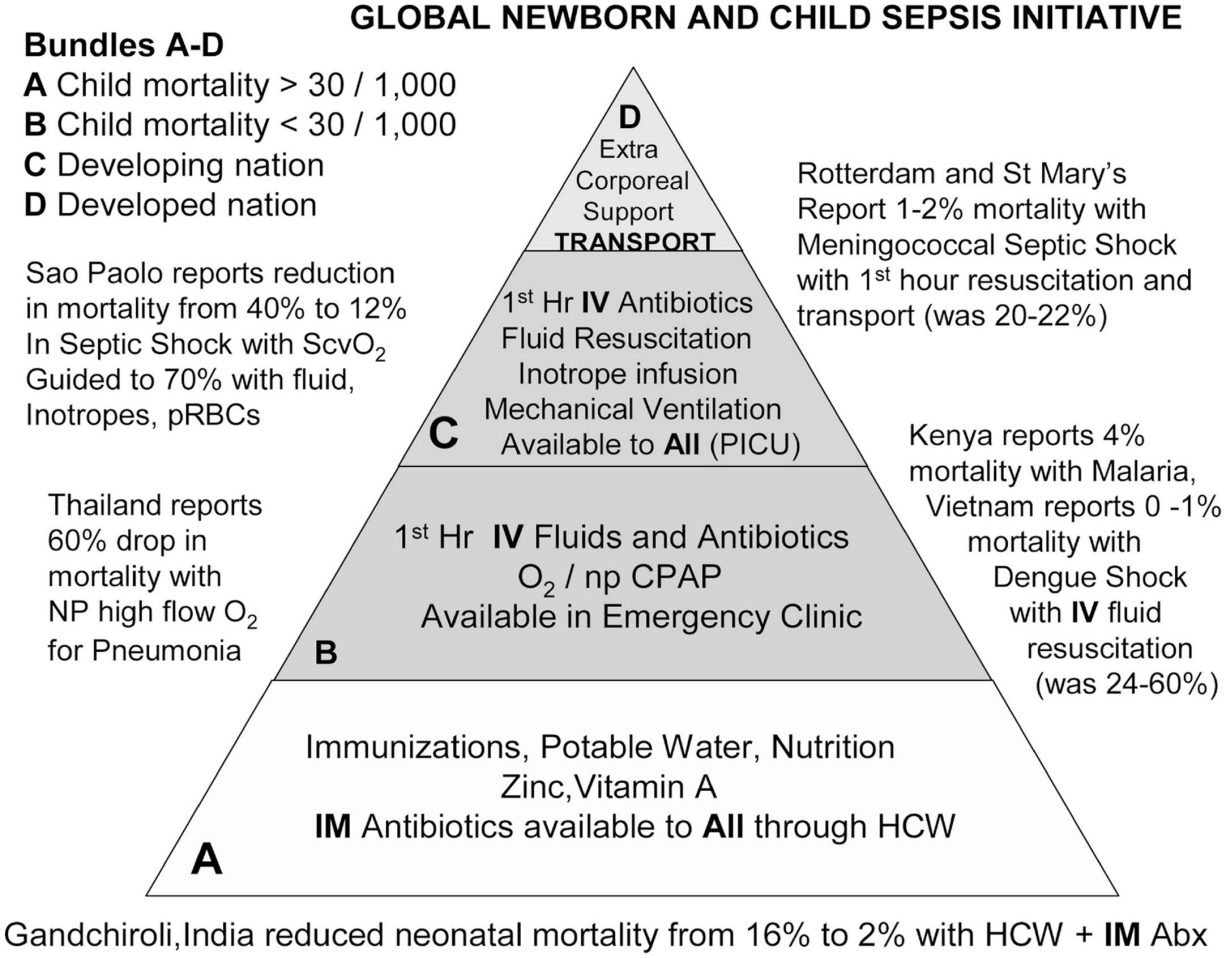
**The sepsis initiative administrative bundles pyramid**. This pyramid demonstrates the administrative recommendations according to levels of health resources from the health resource-scarce (level A) to health resource-abundant (level D). The foundation of care is level A. It is expected to be provided to populations with <5-year child mortality and >30 of 1,000 children. Level B is distinguished from level A by the ability to deliver oxygen and intravenous therapies. It is expected to be provided to populations with <5-year child mortality and <30 of 1,000 children. Category A indicates non-industrialized setting with child mortality rate >30 of 1,000 children; category B indicates non-industrialized setting with child mortality rate <30 of 1,000 children; category C indicates industrialized developing nation; and category D indicates industrialized developed nation. Level C is distinguished from level B by the ability to deliver machine-driven therapy to all. It is expected to be provided in the developing industrialized setting. Level D is distinguished from level C by the presence of an organized transport system and the ability to deliver extracorporeal therapies to all. It is expected to be provided in the developed industrialized setting. Categories A and B are in the non-industrialized setting. Categories C and D are in the industrialized setting. In category A, Bang et al. demonstrated an eightfold reduction in neonatal mortality when intramuscular (*IM*) gentamicin and oral cotrimoxazole were administered by rural healthcare workers. In category B, investigators in Thailand, Vietnam, and Kenya demonstrated that administering high-flow oxygen (*O_2_*) and isotonic intravenous fluid boluses reduced mortality from pneumonia, dengue shock, and severe malaria. In category C, de Oliveira et al. demonstrated a four-fold reduction from septic shock with American College of Critical Care Medicine/Pediatric Advanced Life Support goal-directed therapy. In Rotterdam and London, investigators demonstrated a 10-fold reduction in mortality from purpura and meningococcemia with a transport team and tertiary center care. *IV*, intravenous; *PICU*, pediatric intensive care unit; *npCPAP*, nasopharyngeal continuous positive airway pressure; *HCW*, healthcare worker; *ScvO2*, superior vena cava oxygen saturation; *pRBC*, packed red blood cells; *NP*, nasopharyngeal. Copied with permission from: Kissoon et al. (108).

The Global Pediatric Sepsis Initiative in 2011 categorized best-practice interventions based on under-5-year mortality and developed a web-based educational tool to improve the quality of care for children with sepsis around the globe (see Figure 3) (108). The World Federation of Pediatric Intensive and Critical Care Societies has created both acute and post-acute care bundles for pediatric sepsis with the hopes of providing a conceptual framework for resuscitation management (43).

Still, the direct application of guidelines developed in resource-rich settings to resource-limited settings must be done with caution. The Fluid Expansion as Supportive Therapy (FEAST) trial was a large, multi-center, randomized trial conducted in sub-Saharan Africa that implemented sepsis initiatives with early fluid resuscitation. The trial demonstrated increased 48 hour hospital mortality for the intervention groups (20–40 mL/kg of 5% albumin or 0.9% saline) as compared to the control group (no bolus fluid resuscitation) (109). Although highly criticized, these unexpected findings call to question the assumption that widespread application of resource-rich guidelines to resource-limited settings will yield similar effects. In addition, an attempt to implement the 2008 Surviving Sepsis Campaign guidelines in hospitals across the country of Mongolia highlighted a dramatic shortage of the required hospital facilities, equipment, drugs, and disposable materials necessary for implementation (110). Thus, a thorough assessment of available resources in each local setting is imperative prior to adapting clinical practice to match that of parts of the world with seemingly unlimited resources.

### Critical Resources

In some university and private hospitals in South Africa, Uganda, Kenya, Rwanda, and Namibia, critical care resources are comparable to Western countries. However, in township and district hospitals in sub-Saharan Africa, and South East Asia, ICU care is often very limited (43). What can be found at the district hospital level may be a four- to eight-bed mixed adult and pediatric ICU with one to two nurses and minimal ancillary support. Hospitals often have a very limited supply of patient monitors, mechanical ventilators, necessary disposable materials (ECG stickers, tubing, and so forth), or electricity. Although the WHO recommends oxygen concentrators to be used as the primary oxygen supply for resource-limited settings, many of these countries utilize pressurized oxygen cylinders for oxygen delivery because they are less expensive, do not require electricity or ongoing maintenance, and are easier to use. However, transportation difficulties, exhaustible oxygen supply, and restricted patient use outweigh the initial savings, leading to frequent oxygen shortages (111, 112).

Appropriate supportive care was shown to help reduce many unnecessary deaths in the Ebola epidemic (13). Although often considered part of basic care delivery in resource-rich settings, the availability and sustainability of clean water, reliable electricity, oxygen and compressed air, medical equipment, and support services (radiology, laboratory medicine, microbiology, and transfusion services) are essential for the delivery of critical care. In some areas, lack of basic health care resources, including protective gloves and gowns, intravenous fluids, and simple protocols or guidelines of care, limited the impact of frontline healthcare providers who risked their lives to care for those affected by Ebola (83). As a first step, providing ongoing access to these basic resources may improve survival in many resource-limited settings.

In addition to epidemics, natural and man-made disasters have highlighted the need for an emergent increase in critical care capacity globally. In 2010, the Pediatric Emergency Mass Critical Care Task Force, composed of 36 experts in domains, including medicine, public health, and disaster response, recommended that equipment and supplies be available to triple the typical ICU capacity for at least 10 days during times of disaster or epidemic illness regardless of location (113). The 2014 CHEST consensus statement on critical care in resource-limited settings calls for development of feasibility plans to address a protracted event requiring long-term use of critical care resources and capacity to immediately expand hospital critical care resources by 20–200% depending on the surge response required by the disaster/pandemic (114, 115).

### Healthcare Systems

Despite recent global movement to expand access to health care, the Ebola outbreak was an important reminder to carefully consider two simple questions as outlined by Boozary et al.: What kind of care are people going to access? Is that care worth having, and can it be made better (83)? In order to avoid distrust in healthcare systems by local communities, as evident during the recent Ebola outbreak, the focus on quality of care and its measurement is key in resource-limited settings.

Although it is difficult to define quality improvement measures, recent efforts have been made by Duke et al. using a consensus-based, international approach to identify indicators of hospital care for children and newborns suitable for use in resource-limited settings (116). For example, structural indicators of quality, including availability of intravenous fluids, epinephrine for injection, presence of a triage system, and prioritization and grouping of severely ill children for increased observation were all highly rated quality indicators applicable to care of critically ill children in these settings (116). The quality of care approach should include safety, effectiveness, and care delivery in ways that respect the dignity of individuals in the context of their own “local moral worlds” (117). When people receive care that is unsafe or ineffective, or they are not treated with respect, it is little surprise they will avoid further care (118).

While some might see tradeoffs between interventions to stem current epidemics and investments in health systems for the future, these two notions can coexist. Indeed, building systems that provide high-quality care in crisis can be used to provide effective critical care disease management and chronic care once the epidemic has subsided. The HIV epidemic is an important example of how quality, system-based interventions in times of disease outbreak can serve a country’s healthcare system as a whole.

For example, Rwanda’s AIDS program was characterized not only by efforts to integrate prevention and control, but also by attention to concomitant health problems, such as tuberculosis and malnutrition, to strengthen the healthcare system as a whole. High-level political commitment to equity and service delivery, a clear plan for action, and harnessing funds has been very successful. The country’s ownership of health care spending has facilitated this process. Evidence-based policy making through linkage of research to service and training has helped promote accountability and improve quality of care in Rwanda (119). These interventions enabled a 70% reduction in the under-5-year mortality rate between 2000 and 2011 (119).

### Research Strategies for Clinical Evidence

A major challenge in improving critical care in resource-limited settings as discussed earlier is the lack of evidence to both quantify the burden of disease as well as support interventions for a particular disease. The majority of research studies, in particular in critical care, are conducted in resource-rich settings, targeting the most common and most severe diseases in these settings. The protocol for the Global Burden of Diseases, Injuries, and Risk Factors study in 2013 identified regional and country-specific differences in disease burden (3) making the blind application of effective critical care interventions from resource-rich settings not appropriate while also creating additional financial strain on limited resources. This creates a vicious cycle in which the limitation of resources prevents design and implementation of research initiatives needed to identify which resources are most needed.

The first step in improving outcomes in critical illness is to understand local and regional epidemiology of disease, so that institutions can prioritize future interventions (55). This research requires resources, such as accurate medical record keeping, disease-specific laboratory diagnostics, personnel to gather and analyze data, and local providers to identify potential research subjects. A number of trauma registries in low- and middle-income countries serve as examples for tracking the epidemiology of a disease (120–123), and their ability to contribute to a reduction in mortality has been established (122). Tracking of critical illness proves more challenging because most diagnoses rely on laboratory confirmation rather than clinical history alone (124–126).

Once the local and regional epidemiology of critical illness is understood, targeted interventions and their evaluation represent the next stage. The efficacy of a single intervention depends upon the local environment and resource availability. While relatively low-resource interventions, such as pulse oximetry, have been effective in reducing mortality in resource-limited settings (64), higher resource interventions, such as CPAP, have improved clinical status but not decreased mortality (127). A potential explanation for this discrepancy is that high-resource interventions concentrate available resources on the highest acuity patients, leaving those with milder illness with inadequate monitoring.

Once effective interventions have been identified, efforts are necessary to implement them region- or country-wide. Previously mentioned incorporation of HIV care into outpatient primary care clinics (128) and utilization of various clinical treatment guidelines in resource-limited settings represent an opportunity for improved outcomes on a larger scale.

Once implemented, ongoing monitoring in the form of research is needed to ensure that the interventions are appropriate. Conducting research in resource-limited countries where the population of individuals affected is vulnerable and there is a heightened possibility of exploitation of individuals and communities should comply with important ethical principles (84). These may include use of culturally appropriate forms of informed consent and a local IRB, regulated use of therapies as part of clinical trials to determine effectiveness and validity, avoidance of compassionate use, prevention of exploitation of vulnerable populations, and collaborative partnerships with local communities and stakeholders (84).

In times of disease outbreaks as in the Ebola epidemic, lack of prior approval for research prevents the study of promising interventions, such as vaccines, monoclonal antibodies, and convalescent plasma therapies (129). A potential solution and model for the carefully planned study of future infectious disease outbreaks in times of disaster has been offered by the MSF research ethics committee via pre-approved generic research protocols that are then further defined and approved by the appropriate national authorities once an outbreak occurs (130).

#### Research Agenda for Critical Care in Resource-Poor Settings

Published data on pediatric critical care research from resource-limited countries remains sparse, yet is much needed (18, 73). Reasons for this gap in evidence likely include lack of funding, local critical care providers and researchers, academic mentorship and infrastructure to perform research, and/or barriers to turn available data into publishable research. The limited existing evidence hinders effective and efficient care and advocacy for resource-limited settings.

The agenda for critical care research in resource-limited settings should include more evidence on epidemiology and outcomes. A more accurate estimate of the potential lives saved through pediatric critical care interventions would justify its role in healthcare systems globally (19). Data on critical care capacity, and access to both critical care resources and health care professionals, are essential for health system planning, but generally lacking. Efforts should be made to adjust critical care guidelines from high-income countries to resource-limited settings. Cost-effectiveness analyses of current and proposed critical care practices need to be emphasized (73). Efficacy must be measured and validated for critical care interventions, with limited resources targeted to those practices that save lives, time, and resources. Currently, available critical care mortality prediction tools and triage scoring systems require revalidation in region-specific contexts (131, 132) and low-cost critical care technology is much needed to support critical care in resource-limited settings.

## Conclusion

We affirm that the concept of global justice for all persons needs to be applied to the shortage of pediatric and adult critical care services across the globe. At the core, there is a need to remediate inequalities of access to healthcare as a fundamental human right (133). The injustice of the disparity in healthcare requires tireless patient advocacy for equity of care and increased resources (73). We no longer live in silos; instead, we live in an interconnected world where communications, collaboration, trade, finance, and pollution are all shared (84). The recent Ebola epidemic served as a harsh reminder that in order to value all human life equally, there is an urgent need to bridge resource-rich and resource-limited worlds and build effective, long-lasting partnerships across borders (83).

Critical care has often been deemed inappropriate and of lower priority than primary care efforts and, thus, has additional challenges to overcome in resource-limited settings. However, the lack of prioritization is not justified as discussed above (73). Critics indict clinical technologies and label them as failed strategies, likely due to lack of research in the field, which devalues clinical intervention and diverts the attention from the value of critical care medicine to the overall health of the population (134).

There is no doubt that critical care and public health overlap. An Ebola virus outbreak among a mobile population who carries it to densely populated areas without adequate critical care resources and systems to contain further spread is a huge public health issue (135). The lack of critical care resources in the center of the epidemic and the inability to increase available resources acutely has contributed to the amplification of disease burden, increasing stress on an already deficient public health system.

We argue for a focus on basic pediatric critical care principals with locally appropriate adaptations in collaboration with ministries of health and community members. A concentration on international partnerships, educational initiatives, and research strategies will only help to enhance the specialty of pediatric critical care. Building capacity and evaluating efficacy of critical care interventions in resource-limited settings has the potential to greatly improve the overall outcome of critically ill children by supporting interventions at every level of patient care. The strategies outlined move us toward delivery of an adequate standard of critical care for all children globally and provide an opportunity to dramatically decrease the mortality of children in resource-limited settings.

## Author Contributions

ELT, primary author, wrote the bulk of the sections and edits. KRN wrote sections of the article and participated in editing the paper. SMJ wrote sections of the article and participated in editing the paper. AA wrote sections, participated in editing, and wrote the bibliography. NM wrote sections of the paper and participated in editing and is responsible for the overall content. Thomas Brogan, MD was acknowledged for reading the article and providing important feedback.

## Conflict of Interest Statement

The authors declare that the research was conducted in the absence of any commercial or financial relationships that could be construed as a potential conflict of interest.

## Footnotes

^1^www.pecc-kenya.org

^2^www.openpediatrics.com

^3^www.basics.org

^4^www.sccm.org

## References

[1] Wang H Liddell CA Coates MM Mooney MD Levitz CE Schumacher AE Global, regional, and national levels of neonatal, infant, and under-5 mortality during 1990–2013: a systematic analysis for the global burden of disease study 2013. Lancet (2014) 384(9947):957–79. 10.1016/S0140-6736(14)60497-9 24797572 PMC4165626

[2] Bhutta ZA Black RE Global maternal, newborn, and child health – so near and yet so far. N Engl J Med (2013) 369(23):2226–35. 10.1056/NEJMra1111853 24304052

[3] GBD 2013 Mortality and Causes of Death Collaborators. Global, regional, and national age-sex specific all-cause and cause-specific mortality for 240 causes of death, 1990–2013: a systematic analysis for the global burden of disease study 2013. Lancet (2015) 385(9963):117–71. 10.1016/S0140-6736(14)61682-2 25530442 PMC4340604

[4] Molyneux E Ahmad S Robertson A. Improved triage and emergency care for children reduces inpatient mortality in a resource-constrained setting. Bull World Health Organ (2006) 84(4):314–9. 10.2471/BLT.04.019505 16628305 PMC2627321

[5] Baker T. Pediatric emergency and critical care in low-income countries. Paediatr Anaesth (2009) 19(1):23–7. 10.1111/j.1460-9592.2008.02868.x 19076498

[6] Dunser MW Baelani I Ganbold L. A review and analysis of intensive care medicine in the least developed countries. Crit Care Med (2006) 34(4):1234–42. 10.1097/01.CCM.0000208360.70835.87 16484925

[7] The World Bank (2015). Available from: http://www.worldbank.org/en/topic/ebola/brief/global-ebola-response-resource-tracking

[8] Thomas MR Smith G Ferreira FHG Evans D Maliszewska M Cruz M The economic impact of Ebola on sub-Saharan Africa: updated estimates for 2015. World Bank Group (2015). Available from: http://documents.worldbank.org/curated/en/2015/01/23831803/economic-impact-ebola-sub-saharan-africa-updated-estimates-2015

[9] Centers for Disease Control and Prevention. (2016). Available from: http://www.cdc.gov/vhf/ebola/outbreaks/2014-west-africa/case-counts.html

[10] McElroy AK Erickson BR Flietstra TD Rollin PE Nichol ST Towner JS Biomarker correlates of survival in pediatric patients with Ebola virus disease. Emerg Infect Dis (2014) 20(10):1683–90. 10.3201/eid2010.140430 25279581 PMC4193175

[11] WHO Ebola Response Team Agua-Agum J Ariyarajah A Blake IM Cori A Donnelly CA Ebola virus disease among children in West Africa. N Engl J Med (2015) 372(13):1274–7. 10.1056/NEJMc1415318 25806936 PMC4393247

[12] Eriksson CO Uyeki TM Christian MD King MA Braner DA Kanter RK Care of the child with Ebola virus disease. Pediatr Crit Care Med (2015) 16(2):97–103. 10.1097/PCC.0000000000000358 25647119

[13] Lamontagne F Clément C Fletcher T Jacob ST Fischer WA II Fowler RA Doing today’s work superbly well – treating Ebola with current tools. N Engl J Med (2014) 371(17):1565–6. 10.1056/NEJMp1411310 25251518

[14] Wheeler DS Wong HR Shanley TP, editors. Science and Practice of Pediatric Critical Care Medicine. London: Springer (2009). p. 1–28.

[15] Pediatric section task force on admission and discharge criteria, society of critical care medicine in conjunction with the American college of critical care medicine and the committee on hospital care of the American academy of pediatrics. Guidelines for developing admission and discharge policies for the pediatric intensive care unit. Crit Care Med (1999) 27(4):843–5. 10321680

[16] Task force of the American college of critical care medicine, society of critical care medicine. Guidelines for intensive care unit admission, discharge, and triage. Crit Care Med (1999) 27(3):633–8. 10.1097/00003246-199903000-00048 10199547

[17] Scales DC Rubenfeld GD The Organization of Critical Care: An Evidence-Based Approach to Improving Quality. Respiratory Medicine. New York: Humana Press (2014).

[18] Adhikari NK Fowler RA Bhagwanjee S Rubenfeld GD. Critical care and the global burden of critical illness in adults. Lancet (2010) 376(9749):1339–46. 10.1016/S0140-6736(10)60446-1 20934212 PMC7136988

[19] Murthy S Sayeed SA Adhikari KJ Critical care in low-resource settings. In: Scales DC Rubenfeld GD, editors. The Organization of Critical Care. New York: Springer Science+Business (2014). p. 247–60.

[20] Widdowson MA Iuliano AD Dawood FS Challenges to global pandemic mortality estimation. Lancet Infect Dis (2014) 14(8):670–2. 10.1016/S1473-3099(14)70835-7 25056013

[21] Simonsen L Spreeuwenberg P Lustig R Taylor RJ Fleming DM Kroneman M Global mortality estimates for the 2009 influenza pandemic from the GLaMOR project: a modeling study. PLoS Med (2013) 10(11):e1001558. 10.1371/journal.pmed.1001558 24302890 PMC3841239

[22] Dawood FS Iuliano AD Reed C Meltzer MI Shay DK Cheng PY Estimated global mortality associated with the first 12 months of 2009 pandemic influenza A H1N1 virus circulation: a modelling study. Lancet Infect Dis (2012) 12(9):687–95. 10.1016/S1473-3099(12)70121-4 22738893

[23] Report of a WHO/International Study Team. Ebola haemorrhagic fever in Sudan, 1976. Bull World Health Organ (1978) 56(2):247–70. 307455 PMC2395561

[24] Metnitz PG Moreno RP Almeida E Jordan B Bauer P Campos RA SAPS 3 – from evaluation of the patient to evaluation of the intensive care unit. Part 1: objectives, methods and cohort description. Intensive Care Med (2005) 31(10):1336–44. 10.1007/s00134-005-2762-6 16132893 PMC1315314

[25] Qureshi AU Ali AS Ahmad TM. Comparison of three prognostic scores (PRISM, PELOD and PIM 2) at pediatric intensive care unit under Pakistani circumstances. J Ayub Med Coll Abbottabad (2007) 19(2):49–53. 18183720

[26] Sankar J Singh A Sankar MJ Joghee S Dewangan S Dubey N. Pediatric index of mortality and PIM2 scores have good calibration in a large cohort of children from a developing country. Biomed Res Int (2014) 2014:907871. 10.1155/2014/907871 25025075 PMC4082889

[27] Taori RN Lahiri KR Tullu MS. Performance of PRISM (pediatric risk of mortality) score and PIM (pediatric index of mortality) score in a tertiary care pediatric ICU. Indian J Pediatr (2010) 77(3):267–71. 10.1007/s12098-010-0031-3 20177831

[28] Wells M Riera-Fanego JF Luyt DK Dance M Lipman J. Poor discriminatory performance of the pediatric risk of mortality (PRISM) score in a South African intensive care unit. Crit Care Med (1996) 24(9):1507–13. 10.1097/00003246-199609000-00013 8797623

[29] Gove S Tamburlini G Molyneux E Whitesell P Campbell H. Development and technical basis of simplified guidelines for emergency triage assessment and treatment in developing countries. WHO integrated management of childhood illness (IMCI) referral care project. Arch Dis Child (1999) 81(6):473–7. 10.1136/adc.81.6.473 10569960 PMC1718149

[30] Kolstad PR Burnham G Kalter HD Kenya-Mugisha N Black RE. The integrated management of childhood illness in western Uganda. Bull World Health Organ (1997) 75(Suppl 1):77–85. 9529720 PMC2486998

[31] Kalter HD Schillinger JA Hossain M Burnham G Saha S de Wit V Identifying sick children requiring referral to hospital in Bangladesh. Bull World Health Organ (1997) 75(Suppl 1):65–75. 9529719 PMC2486991

[32] Perkins BA Zucker JR Otieno J Jafari HS Paxton L Redd SC Evaluation of an algorithm for integrated management of childhood illness in an area of Kenya with high malaria transmission. Bull World Health Organ (1997) 75(Suppl 1):33–42. 9529716 PMC2487004

[33] Weber MW Mulholland EK Jaffar S Troedsson H Gove S Greenwood BM. Evaluation of an algorithm for the integrated management of childhood illness in an area with seasonal malaria in the Gambia. Bull World Health Organ (1997) 75(Suppl 1):25–32. 9529715 PMC2486992

[34] Veirum JE Biai S Jakobsen M Sandström A Hedegaard K Kofoed PE Persisting high hospital and community childhood mortality in an urban setting in Guinea-Bissau. Acta Paediatr (2007) 96(10):1526–30. 10.1111/j.1651-2227.2007.00467.x 17850399

[35] Colvin JD Zaniletti I Fieldston ES Gottlieb LM Raphael JL Hall M Socioeconomic status and in-hospital pediatric mortality. Pediatrics (2013) 131(1):e182–90. 10.1542/peds.2012-1215 23248226

[36] Nolan T Angos P Cunha AJ Muhe L Qazi S Simoes EA Quality of hospital care for seriously ill children in less-developed countries. Lancet (2001) 357(9250):106–10. 10.1016/S0140-6736(00)03542-X 11197397

[37] Irimu G Wamae A Wasunna A Were F Ntoburi S Opiyo N Developing and introducing evidence based clinical practice guidelines for serious illness in Kenya. Arch Dis Child (2008) 93(9):799–804. 10.1136/adc.2007.126508 18719161 PMC2654066

[38] Ayieko P Ntoburi S Wagai J Opondo C Opiyo N Migiro S A multifaceted intervention to implement guidelines and improve admission paediatric care in Kenyan district hospitals: a cluster randomised trial. PLoS Med (2011) 8(4):e1001018. 10.1371/journal.pmed.1001018 21483712 PMC3071366

[39] Reyburn H Mwakasungula E Chonya S Mtei F Bygbjerg I Poulsen A Clinical assessment and treatment in paediatric wards in the north-east of the united republic of Tanzania. Bull World Health Organ (2008) 86(2):132–9. 10.2471/BLT.07.041723 18297168 PMC2647389

[40] English M Esamai F Wasunna A Were F Ogutu B Wamae A Assessment of inpatient paediatric care in first referral level hospitals in 13 districts in Kenya. Lancet (2004) 363(9425):1948–53. 10.1016/S0140-6736(04)16408-8 15194254

[41] English M Esamai F Wasunna A Were F Ogutu B Wamae A Delivery of paediatric care at the first-referral level in Kenya. Lancet (2004) 364(9445):1622–9. 10.1016/S0140-6736(04)17318-2 15519635

[42] WHO, editor. IMAI District Clinician Manual: Hospital Care for Adolescents and Adults. Guidelines for the Management of Illnesses with Limited Resources. Geneva: WHO (2012).

[43] Dünser MW Festic E Dondorp A Kissoon N Ganbat T Kwizera A Recommendations for sepsis management in resource-limited settings. Intensive Care Med (2012) 38(4):557–74. 10.1007/s00134-012-2468-5 22349419 PMC3307996

[44] Gosselin RA Maldonado A Elder G. Comparative cost-effectiveness analysis of two MSF surgical trauma centers. World J Surg (2010) 34(3):415–9. 10.1007/s00268-009-0230-0 19771466 PMC2816808

[45] Busse H Azazh A Teklu S Tupesis JP Woldetsadik A Wubben RJ Creating change through collaboration: a twinning partnership to strengthen emergency medicine at Addis Ababa University/Tikur Anbessa specialized hospital – a model for international medical education partnerships. Acad Emerg Med (2013) 20(12):1310–8. 10.1111/acem.12265 24341587

[46] WHO, editor. Pocket Book of Hospital Care for Children: Guidelines for the Management of Common Childhood Illnesses. 2nd ed Geneva: WHO Press (2013). 24006557

[47] Li MY Puspita R Duke T Agung FH Hegar B Pritasari K Implementation in Indonesia of the WHO pocket book of hospital care for children. Paediatr Int Child Health (2014) 34(2):84–91. 10.1179/2046905513Y.0000000075 24090481

[48] Li MY Kelly J Subhi R Were W Duke T. Global use of the WHO pocket book of hospital care for children. Paediatr Int Child Health (2013) 33(1):4–17. 10.1179/2046905512Y.0000000017 23485489

[49] Ahmed T Ali M Ullah MM Choudhury IA Haque ME Salam MA Mortality in severely malnourished children with diarrhoea and use of a standardised management protocol. Lancet (1999) 353(9168):1919–22. 10.1016/S0140-6736(98)07499-6 10371570

[50] Ashworth A Chopra M McCoy D Sanders D Jackson D Karaolis N WHO guidelines for management of severe malnutrition in rural South African hospitals: effect on case fatality and the influence of operational factors. Lancet (2004) 363(9415):1110–5. 10.1016/S0140-6736(04)15894-7 15064029

[51] Gathara D Opiyo N Wagai J Ntoburi S Ayieko P Opondo C Quality of hospital care for sick newborns and severely malnourished children in Kenya: a two-year descriptive study in 8 hospitals. BMC Health Serv Res (2011) 11:307. 10.1186/1472-6963-11-307 22078071 PMC3236590

[52] English M Nzinga J Mbindyo P Ayieko P Irimu G Mbaabu L Explaining the effects of a multifaceted intervention to improve inpatient care in rural Kenyan hospitals – interpretation based on retrospective examination of data from participant observation, quantitative and qualitative studies. Implement Sci (2011) 6:124 10.1186/1748-5908-6-124 22132875 PMC3248845

[53] de Savigny D Adam T editors. Systems Thinking for Health System Strengthening. Alliance for Health Policy and Systems Research. Geneva: World Health Organization (2009).

[54] Jayaram R Ramakrishnan N Cost of intensive care in India. Indian J Crit Care Med (2008) 12(2):55–61. 10.4103/0972-5229.42558 19742248 PMC2738307

[55] Firth P Ttendo S Intensive care in low-income countries – a critical need. N Engl J Med (2012) 367(21):1974–6. 10.1056/NEJMp1204957 23171093

[56] Shann F Role of intensive care in countries with a high child mortality rate. Pediatr Crit Care Med (2011) 12(1):114–5. 10.1097/PCC.0b013e3181d504cd 21209577

[57] WHO. CHOosing Interventions that Are Cost Effective (WHO-CHOICE). Geneva: WHO (2010).

[58] Morel CM Lauer JA Evans DB. Cost effectiveness analysis of strategies to combat malaria in developing countries. BMJ (2005) 331(7528):1299. 10.1136/bmj.38639.702384.AE 16282381 PMC1298848

[59] Adam T Lim SS Mehta S Bhutta ZA Fogstad H Mathai M Cost effectiveness analysis of strategies for maternal and neonatal health in developing countries. BMJ (2005) 331(7525):1107. 10.1136/bmj.331.7525.1107 16282407 PMC1283271

[60] WHO. (2015). Available from: http://www.who.int/choice/interventions/en/

[61] Gosselin RA Thind A Bellardinelli A. Cost/DALY averted in a small hospital in Sierra Leone: what is the relative contribution of different services? World J Surg (2006) 30(4):505–11. 10.1007/s00268-005-0609-5 16528459

[62] Gosselin RA Heitto M. Cost-effectiveness of a district trauma hospital in Battambang, Cambodia. World J Surg (2008) 32(11):2450–3. 10.1007/s00268-008-9708-4 18716830

[63] McCord C Chowdhury Q. A cost effective small hospital in Bangladesh: what it can mean for emergency obstetric care. Int J Gynaecol Obstet (2003) 81(1):83–92. 10.1016/S0020-7292(03)00072-9 12676406

[64] Duke T Wandi F Jonathan M Matai S Kaupa M Saavu M Improved oxygen systems for childhood pneumonia: a multihospital effectiveness study in Papua New Guinea. Lancet (2008) 372(9646):1328–33. 10.1016/S0140-6736(08)61164-2 18708248

[65] Williams D Flory S King R Thornton M Dingley J. A low oxygen consumption pneumatic ventilator for emergency construction during a respiratory failure pandemic. Anaesthesia (2010) 65(3):235–42. 10.1111/j.1365-2044.2009.06207.x 20064146 PMC7161812

[66] Beringer RM Eltringham RJ. The Glostavent: evolution of an anaesthetic machine for developing countries. Anaesth Intensive Care (2008) 36(3):442–8. 18564808 10.1177/0310057X0803600317

[67] Parikh CR Karnad DR. Quality, cost, and outcome of intensive care in a public hospital in Bombay, India. Crit Care Med (1999) 27(9):1754–9. 10.1097/00003246-199901001-00033 10507594

[68] Kahn JM Angus DC. Health policy and future planning for survivors of critical illness. Curr Opin Crit Care (2007) 13(5):514–8. 10.1097/MCC.0b013e3282efb7c9 17762228

[69] Caney S Justice Beyond Borders. Oxford: Oxford University Press (2005).

[70] Engelhardt HT Jr. Critical care: why there is no global bioethics. Curr Opin Crit Care (2005) 11(6):605–9. 10.1097/01.ccx.0000184166.10121.c1 16292068

[71] Engelhardt HT Jr. Critical care: why there is no global bioethics. J Med Philos (1998) 23(6):643–51. 10.1076/jmep.23.6.643.2555 10190846

[72] Towey RM Ojara S. Intensive care in the developing world. Anaesthesia (2007) 62(Suppl 1):32–7. 10.1111/j.1365-2044.2007.05295.x 17937711

[73] Riviello ED Letchford S Achieng L Newton MW. Critical care in resource-poor settings: lessons learned and future directions. Crit Care Med (2011) 39(4):860–7. 10.1097/CCM.0b013e318206d6d5 21297458

[74] von Saint André-von Arnim A Brogan TV Hertzig J Kim K Wurm G Roberts J Intensive care for infants and children in Haiti in April 2010. Pediatr Crit Care Med (2011) 12(4):393–7. 10.1097/PCC.0b013e318219268d 21478800

[75] Argent AC Ahrens J Morrow BM Reynolds LG Hatherill M Salie S Pediatric intensive care in South Africa: an account of making optimum use of limited resources at the Red Cross War Memorial Children’s Hospital. Pediatr Crit Care Med (2014) 15(1):7–14. 10.1097/PCC.0000000000000029 24389708

[76] Basnet S Adhikari N Koirala J. Challenges in setting up pediatric and neonatal intensive care units in a resource-limited country. Pediatrics (2011) 128(4):e986–92. 10.1542/peds.2010-3657 21930539

[77] Basnet S Shrestha S Ghimire A Timila D Gurung J Karki U Development of a PICU in Nepal: the experience of the first year. Pediatr Crit Care Med (2014) 15(7):e314–20. 10.1097/PCC.0000000000000201 25080149

[78] Baker T. Critical care in low-income countries. Trop Med Int Health (2009) 14(2):143–8. 10.1111/j.1365-3156.2008.02202.x 19207174

[79] Molyneux E. Emergency care for children in resource-constrained countries. Trans R Soc Trop Med Hyg (2009) 103(1):11–5. 10.1016/j.trstmh.2008.07.002 18768191

[80] Wallis PA Gottschalk SB Wood D Bruijns S de Vries S Balfour C The cape triage score – a triage system for South Africa. S Afr Med J (2006) 96(1):53–6. 16440113

[81] Bruijns SR Wallis LA Burch VC. A prospective evaluation of the cape triage score in the emergency department of an urban public hospital in South Africa. Emerg Med J (2008) 25(7):398–402. 10.1136/emj.2007.051177 18573947

[82] Khilnani P Chhabra R. Transport of critically ill children: how to utilize resources in the developing world. Indian J Pediatr (2008) 75(6):591–8. 10.1007/s12098-008-0115-5 18759088

[83] Boozary AS Farmer PE Jha AK The Ebola outbreak, fragile health systems, and quality as a cure. JAMA (2014) 312(18):1859–60. 10.1001/jama.2014.14387 25285459

[84] Rid A Emanuel EJ Why should high-income countries help combat Ebola? JAMA (2014) 312(13):1297–8. 10.1001/jama.2014.12869 25210838

[85] Okafor UV. Challenges in critical care services in Sub-Saharan Africa: perspectives from Nigeria. Indian J Crit Care Med (2009) 13(1):25–7. 10.4103/0972-5229.53112 19881176 PMC2772254

[86] Physicians for Human Rights. An Action Plan to Prevent Brain Drain: Building Equitable Health Systems in Africa (2004). Available from: http://physiciansforhumanrights.org/library/reports/action-plan-to-prevent-brain-drain-africa-2004.html

[87] Mullan F. The metrics of the physician brain drain. N Engl J Med (2005) 353(17):1810–8. 10.1056/NEJMsa050004 16251537

[88] WHO. (2015). Available from: http://www.who.int/hrh/migration/code/WHO_global_code_of_practice_EN.pdf

[89] Tankwanchi AB Vermund SH Perkins DD. Monitoring Sub-Saharan African physician migration and recruitment post-adoption of the WHO code of practice: temporal and geographic patterns in the United States. PLoS One (2015) 10(4):e0124734. 10.1371/journal.pone.0124734 25875010 PMC4395332

[90] Du B Xi X Chen D Peng J China Critical Care Clinical Trial Group (CCCCTG). Clinical review: critical care medicine in mainland China. Crit Care (2010) 14(1):206. 10.1186/cc8222 20236446 PMC2875498

[91] Prayag S ICUs worldwide: critical care in India. Crit Care (2002) 6(6):479–80. 10.1186/cc1544 12493068 PMC153430

[92] Mustafa I. Intensive care in developing countries in the Western Pacific. Curr Opin Crit Care (2004) 10(4):304–9. 10.1097/01.ccx.0000132653.52131.fc 15258503

[93] Cousin DB Barrett H Bion JF Cohen NH Crisis in critical care: training and certifying future intensivists. Curr Opin Anaesthesiol (2006) 19(2):107–10. 10.1097/01.aco.0000192766.04582.39 16552214

[94] Walker D Cohen S Fritz J Olvera M Lamadrid-Figueroa H Cowan JG Team training in obstetric and neonatal emergencies using highly realistic simulation in Mexico: impact on process indicators. BMC Pregnancy Childbirth (2014) 14(1):367. 10.1186/s12884-014-0367-1 25409895 PMC4243314

[95] Goudar SS Somannavar MS Clark R Lockyer JM Revankar AP Fidler HM Stillbirth and newborn mortality in India after helping babies breathe training. Pediatrics (2013) 131(2):e344–52. 10.1542/peds.2012-2112 23339215

[96] Ellenby MS Marcin JP. The role of telemedicine in pediatric critical care. Crit Care Clin (2015) 31(2):275–90. 10.1016/j.ccc.2014.12.006 25814454

[97] Zachariah R Bienvenue B Ayada L Manzi M Maalim A Engy E Practicing medicine without borders: tele-consultations and tele-mentoring for improving paediatric care in a conflict setting in Somalia? Trop Med Int Health (2012) 17(9):1156–62. 10.1111/j.1365-3156.2012.03047.x 22845678

[98] Martinez Garcia D Bonnardot L Olson D Roggeveen H Karsten J Moons P A retrospective analysis of pediatric cases handled by the MSF tele-expertise system. Front Public Health (2014) 2:266. 10.3389/fpubh.2014.00266 25538935 PMC4260224

[99] Bang AT Bang RA Baitule SB Reddy MH Deshmukh MD. Effect of home-based neonatal care and management of sepsis on neonatal mortality: field trial in rural India. Lancet (1999) 354(9194):1955–61. 10.1016/S0140-6736(99)03046-9 10622298

[100] Rosseel P Trelles M Guilavogui S Ford N Chu K. Ten years of experience training non-physician anesthesia providers in Haiti. World J Surg (2010) 34(3):453–8. 10.1007/s00268-009-0192-2 19655194

[101] Chamberlain S Stolz U Dreifuss B Nelson SW Hammerstedt H Andinda J Mortality related to acute illness and injury in rural Uganda: task shifting to improve outcomes. PLoS One (2015) 10(4):e0122559. 10.1371/journal.pone.0122559 25849960 PMC4388510

[102] Kredo T Adeniyi FB Bateganya M Pienaar ED. Task shifting from doctors to non-doctors for initiation and maintenance of antiretroviral therapy. Cochrane Database Syst Rev (2014) 7:CD007331. 10.1002/14651858.CD007331.pub3 24980859 PMC11214583

[103] Vukoja M Riviello E Gavrilovic S Adhikari NK Kashyap R Bhagwanjee S A survey on critical care resources and practices in low- and middle-income countries. Glob Heart (2014) 9(3):337–42.e1. 10.1016/j.gheart.2014.08.002 25667185

[104] Twomey M Cheema B Buys H Cohen K de Sa A Louw P Vital signs for children at triage: a multicentre validation of the revised South African triage scale (SATS) for children. S Afr Med J (2013) 103(5):304–8. 10.7196/samj.6877 23971119

[105] Steele C. Helping babies breathe around the world. J Obstet Gynecol Neonatal Nurs (2013) 42(2):243–6. 10.1111/1552-6909.12019 23373533

[106] WHO. In: W.W.G.o.C.C.i.L.-R. Settings, editor. Clinical Management of Adult Patients with Complications of Pandemic Influenza A (H1N1) 2009: Emergency Guidelines for the Management of Patients with Severe Respiratory Distress and Shock in District Hospitals in Limited-Resource Settings. Geneva: WHO Press (2010).

[107] WHO. Clinical Management of Severe Respirtatory Infections When Novel Coronavirus Is Suspected: What to Do and What Not to Do.

[108] Kissoon N Carcillo JA Espinosa V Argent A Devictor D Madden M World federation of pediatric intensive care and critical care societies: global sepsis initiative. Pediatr Crit Care Med (2011) 12(5):494–503. 10.1097/PCC.0b013e318207096c 21897156

[109] Maitland K Kiguli S Opoka RO Engoru C Olupot-Olupot P Akech SO Mortality after fluid bolus in African children with severe infection. N Engl J Med (2011) 364(26):2483–95. 10.1056/NEJMoa1101549 21615299

[110] Bataar O Lundeg G Tsenddorj G Jochberger S Grander W Baelani I Nationwide survey on resource availability for implementing current sepsis guidelines in Mongolia. Bull World Health Organ (2010) 88(11):839–46. 10.2471/BLT.10.077073 21076565 PMC2971517

[111] Belle J Cohen H Shindo N Lim M Velazquez-Berumen A Ndihokubwayo JB Influenza preparedness in low-resource settings: a look at oxygen delivery in 12 African countries. J Infect Dev Ctries (2010) 4(7):419–24. 10.3855/jidc.859 20818088

[112] Dobson MB. Oxygen concentrators and cylinders. Int J Tuberc Lung Dis (2001) 5(6):520–3. 11409577

[113] Bohn D Kanter RK Burns J Barfield WD Kissoon N Task Force for Pediatric Emergency Mass Critical Care. Supplies and equipment for pediatric emergency mass critical care. Pediatr Crit Care Med (2011) 12(6 Suppl):S120–7. 10.1097/PCC.0b013e318234a6b9 22067920 PMC4561174

[114] Geiling J Burkle FM Jr Amundson D Dominguez-Cherit G Gomersall CD Lim ML Resource-poor settings: infrastructure and capacity building: care of the critically ill and injured during pandemics and disasters: CHEST consensus statement. Chest (2014) 146(4 Suppl):156S–67S. 10.1378/chest.14-0744 25144337 PMC6679686

[115] Hick JL Einav S Hanfling D Kissoon N Dichter JR Devereaux AV Surge capacity principles: care of the critically ill and injured during pandemics and disasters: CHEST consensus statement. Chest (2014) 146(4 Suppl):e1S–e16S. 10.1378/chest.14-0733 25144334

[116] Ntoburi S Hutchings A Sanderson C Carpenter J Weber M English M Development of paediatric quality of inpatient care indicators for low-income countries – a Delphi study. BMC Pediatr (2010) 10:90. 10.1186/1471-2431-10-90 21144065 PMC3022793

[117] Kleinman A Kleinman J. Suffering and its professional transformation: toward an ethnography of interpersonal experience. Cult Med Psychiatry (1991) 15(3):275–301. 10.1007/BF00046540 1935180

[118] Berendes S Heywood P Oliver S Garner P. Quality of private and public ambulatory health care in low and middle income countries: systematic review of comparative studies. PLoS Med (2011) 8(4):e1000433. 10.1371/journal.pmed.1000433 21532746 PMC3075233

[119] Farmer PE Nutt CT Wagner CM Sekabaraga C Nuthulaganti T Weigel JL Reduced premature mortality in Rwanda: lessons from success. BMJ (2013) 346:f65 10.1136/bmj.f65 23335479 PMC3548616

[120] Schultz CR Ford HR Cassidy LD Shultz BL Blanc C King-Schultz LW Development of a hospital-based trauma registry in Haiti: an approach for improving injury surveillance in developing and resource-poor settings. J Trauma (2007) 63(5):1143–54. 10.1097/TA.0b013e31815688e3 17993964

[121] Kobusingye OC Lett RR. Hospital-based trauma registries in Uganda. J Trauma (2000) 48(3):498–502. 10.1097/00005373-200003000-00022 10744292

[122] O’Reilly GM Joshipura M Cameron PA Gruen R. Trauma registries in developing countries: a review of the published experience. Injury (2013) 44(6):713–21. 10.1016/j.injury.2013.02.003 23473265

[123] malERA Consultative Group on Diagnoses and Diagnostics. A research agenda for malaria eradication: diagnoses and diagnostics. PLoS Med (2011) 8(1):e1000396. 10.1371/journal.pmed.1000396 21311583 PMC3026696

[124] Forgie IM Campbell H Lloyd-Evans N Leinonen M O’Neill KP Saikku P Etiology of acute lower respiratory tract infections in children in a rural community in The Gambia. Pediatr Infect Dis J (1992) 11(6):466–73. 10.1097/00006454-199206000-00009 1608684

[125] Weber MW Mulholland EK Greenwood BM. Respiratory syncytial virus infection in tropical and developing countries. Trop Med Int Health (1998) 3(4):268–80. 10.1046/j.1365-3156.1998.00213.x 9623927

[126] Das CK Mirdha BR Singh S Seth R Bagga A Lodha R Use of induced sputum to determine the prevalence of *Pneumocystis jirovecii* in immunocompromised children with pneumonia. J Trop Pediatr (2014) 60(3):216–22. 10.1093/tropej/fmt112 24425204

[127] Wilson PT Morris MC Biagas KV Otupiri E Moresky RT. A randomized clinical trial evaluating nasal continuous positive airway pressure for acute respiratory distress in a developing country. J Pediatr (2013) 162(5):988–92. 10.1016/j.jpeds.2012.10.022 23164308

[128] Topp SM Chipukuma JM Giganti M Mwango LK Chiko LM Tambatamba-Chapula B Strengthening health systems at facility-level: feasibility of integrating antiretroviral therapy into primary health care services in Lusaka, Zambia. PLoS One (2010) 5(7):e11522. 10.1371/journal.pone.0011522 20644629 PMC2903482

[129] Bausch DG Sprecher AG Jeffs B Boumandouki P. Treatment of Marburg and Ebola hemorrhagic fevers: a strategy for testing new drugs and vaccines under outbreak conditions. Antiviral Res (2008) 78(1):150–61. 10.1016/j.antiviral.2008.01.152 18336927

[130] Schopper D Upshur R Matthys F Singh JA Bandewar SS Ahmad A Research ethics review in humanitarian contexts: the experience of the independent ethics review board of Medecins Sans Frontieres. PLoS Med (2009) 6(7):e1000115 10.1371/journal.pmed.1000115 19636356 PMC2708346

[131] Aggarwal AN Sarkar P Gupta D Jindal SK. Performance of standard severity scoring systems for outcome prediction in patients admitted to a respiratory intensive care unit in North India. Respirology (2006) 11(2):196–204. 10.1111/j.1440-1843.2006.00828.x 16548906

[132] Wheeler I Price C Sitch A Banda P Kellett J Nyirenda M Early warning scores generated in developed healthcare settings are not sufficient at predicting early mortality in Blantyre, Malawi: a prospective cohort study. PLoS One (2013) 8(3):e59830 10.1371/journal.pone.0059830 23555796 PMC3612104

[133] Farmer P Campos NG. Rethinking medical ethics: a view from below. Dev World Bioeth (2004) 4(1):17–41. 10.1111/j.1471-8731.2004.00065.x 15086372

[134] Wise PH. Confronting racial disparities in infant mortality: reconciling science and politics. Am J Prev Med (1993) 9(6 Suppl):7–16. 8123287

[135] Fowler RA Fletcher T Fischer WA II Lamontagne F Jacob S Brett-Major D Caring for critically ill patients with ebola virus disease. Perspectives from West Africa. Am J Respir Crit Care Med (2014) 190(7):733–7. 10.1164/rccm.201408-1514CP 25166884

